# How to learn with intentional mistakes: NoisyEnsembles to overcome poor tissue quality for deep learning in computational pathology

**DOI:** 10.3389/fmed.2022.959068

**Published:** 2022-08-29

**Authors:** Robin S. Mayer, Steffen Gretser, Lara E. Heckmann, Paul K. Ziegler, Britta Walter, Henning Reis, Katrin Bankov, Sven Becker, Jochen Triesch, Peter J. Wild, Nadine Flinner

**Affiliations:** ^1^Dr. Senckenberg Institute of Pathology, University Hospital Frankfurt, Frankfurt am Main, Germany; ^2^Department of Gynecology and Obstetrics, University Hospital Frankfurt, Frankfurt am Main, Germany; ^3^Frankfurt Institute for Advanced Studies (FIAS), Frankfurt am Main, Germany; ^4^Wildlab, University Hospital Frankfurt MVZ GmbH, Frankfurt am Main, Germany; ^5^Frankfurt Cancer Institute (FCI), Frankfurt am Main, Germany; ^6^University Cancer Center (UCT) Frankfurt-Marburg, Frankfurt am Main, Germany

**Keywords:** ovarian cancer, tissue quality, quality control, deep learning, machine learning, computational pathology, ensemble learning, data perturbation

## Abstract

There is a lot of recent interest in the field of computational pathology, as many algorithms are introduced to detect, for example, cancer lesions or molecular features. However, there is a large gap between artificial intelligence (AI) technology and practice, since only a small fraction of the applications is used in routine diagnostics. The main problems are the transferability of convolutional neural network (CNN) models to data from other sources and the identification of uncertain predictions. The role of tissue quality itself is also largely unknown. Here, we demonstrated that samples of the TCGA ovarian cancer (TCGA-OV) dataset from different tissue sources have different quality characteristics and that CNN performance is linked to this property. CNNs performed best on high-quality data. Quality control tools were partially able to identify low-quality tiles, but their use did not increase the performance of the trained CNNs. Furthermore, we trained NoisyEnsembles by introducing label noise during training. These NoisyEnsembles could improve CNN performance for low-quality, unknown datasets. Moreover, the performance increases as the ensemble become more consistent, suggesting that incorrect predictions could be discarded efficiently to avoid wrong diagnostic decisions.

## Introduction

Pathology has always been responsible for the characterization of tissue samples, while nowadays digital pathology transforms this discipline from a semi-quantitative to a quantitative one (1). In addition to algorithms for quantification, convolutional neural networks (CNN) can recognize and classify tumorous tissue (2, 3), predict molecular properties (4–7), and segment morphological structures (8). However, most of these algorithms are not yet used in routine diagnostics because they do not meet regulatory approvals, leaving a large gap between AI in science and clinics. It was not until 2021 that the first AI algorithm in computational pathology was approved by the Food and Drug Administration (FDA) (9). A major problem in overcoming this “translational valley of death” is to ensure the reproducibility and generalizability of the developed products (10) by defining appropriate test datasets (11). All of these applications and algorithms depend, of course, on the digitalized histomorphological whole slide image (WSI) used in training and during application. Therefore, it is important to understand exactly how algorithms respond to limitations, such as poor tissue quality, and how to avoid negative influences before approval.

Recently, a first stress test for a prostate cancer algorithm investigated the influence of various artificially introduced artifacts and showed that, depending on the severity, all tested artifacts can lead to a loss of performance (12). In particular, focus, strong JPEG compression, dark spots, and brightness or contrast changes most strongly affected the results in this study. Consistent with these findings, JPEG compression has also been studied in breast cancer, showing that 85% or a ratio of 24:1 is possible without significant performance loss (13, 14). The out-of-focus effect has also been studied and can be detected using Deep Learning (15–18). In line with the stress test, it is also shown for breast cancer images that performance decreases with generated and real blur effects (16).

However, the most commonly studied artifact in Computational Pathology is the color appearance of H&E (hematoxylin and eosin)-stained WSIs and it is well-known that differences between datasets can drastically affect the transferability of Deep Learning algorithms. Here, there are several tools to align color profiles in different datasets and improve the transferability of CNNs that depend on different methods: Traditional methods use stain separation and preserve the structure completely (19, 20), while newer methods use Deep Learning-based style transfers to normalize colors (21, 22).

As it becomes more apparent that tissue quality and image artifacts strongly influence the results of automated analyses, and as more WSIs become available to develop these algorithms, it is important to provide automated quality control for all images used. Therefore, the first global quality control tools have appeared: HistoQC (23) uses Deep Learning to identify artifact-free regions in WSIs and provides a combination of image metrics to detect outliers; agreement with pathologists is in the range of ~95% for 450 WSIs from TCGA. PathProfiler (24) also evaluates WSIs for usability using Deep Learning and achieves a correlation of 0.89 to pathologists for prostate WSIs from the ProMPT study.

However, it is not entirely clear to what extent the use of such tools improves the performance of CNNs. It also needs to be clarified whether poor-quality slides can be used for algorithm development. In addition, methods are needed to distinguish certain and uncertain predictions when using the trained algorithms. Therefore, we introduce NoisyEnsembles, which can improve the transferability of algorithms trained on high-quality data to unseen data of lower quality. Taking into account the consistency of the ensemble, low-quality tiles could be sorted out and the performance further improved.

## Materials and methods

### Datasets, annotation, and scoring

WSIs from the TCGA-OV dataset (*n* = 101 DX slides, Supplementary Table S1) were downloaded and used for training, validation, and testing as described in this manuscript. In addition, WSIs from the UKF (University Clinic Frankfurt, *n* = 41) were used as an external test dataset. These tissue samples were provided by the University Cancer Center Frankfurt (UCT). Written informed consent was obtained from all patients and the study was approved by the Institutional Review Boards of the UCT and the Ethical Committee at the University Hospital Frankfurt (project-number: UCT-5-2021).

First, all WSIs from the TCGA-OV dataset were evaluated by a pathologist (S.G.) for (i) their overall quality and (ii) their difficulty in distinguishing cancerous from non-cancerous regions. Staining intensity, contrast, and tumor viability were considered when evaluating tissue quality. Excessively strong or weak staining that made it difficult to distinguish individual cells was considered a sign of poor quality. Weak cytoplasmic nuclear contrast was another sign of poor quality. Cases in which specimens had large areas of necrosis or only a small amount of vital tumor were also considered difficult to evaluate. Pathologist scores for quality and rating are given in Supplementary Table S1, example tiles of low, median, and good quality are shown in Supplementary Figure S1.

All WSIs from the TCGA-OV dataset were annotated by the same pathologist (S.G.) using QuPath's (25) annotation tools (polygon, wand, and brush) to delineate cancerous regions. The remaining tissue was then classified as non-cancerous. No regions were excluded due to bad quality. WSIs of the UKF dataset were annotated by a pathologist using the Sysmex CaseViewer software to place circles (diameter: 500 μm) in the regions of interest (cancer and non-cancer).

Then, 40 image patches per patient (512 x 512 pixels, 0.27 μm/pixel) were extracted from the labeled areas. To ensure that we did not have mislabeled images in our datasets, which could occur, for example, due to non-accurate annotations in border regions, we trained CNNs to predict the appropriate labeling, and 188/94 incorrectly predicted tiles were rechecked by a pathologist (S.G.) and relabeled if necessary. In addition, 98/188 tiles of the TCGA-OV and 29/94 tiles of the UKF dataset were removed completely, because it was not possible to determine the appropriate label (e.g., because of extreme blur or only some single cells were left on the image).

### CNN training and NoisyEnsembles

**Figure 1:**
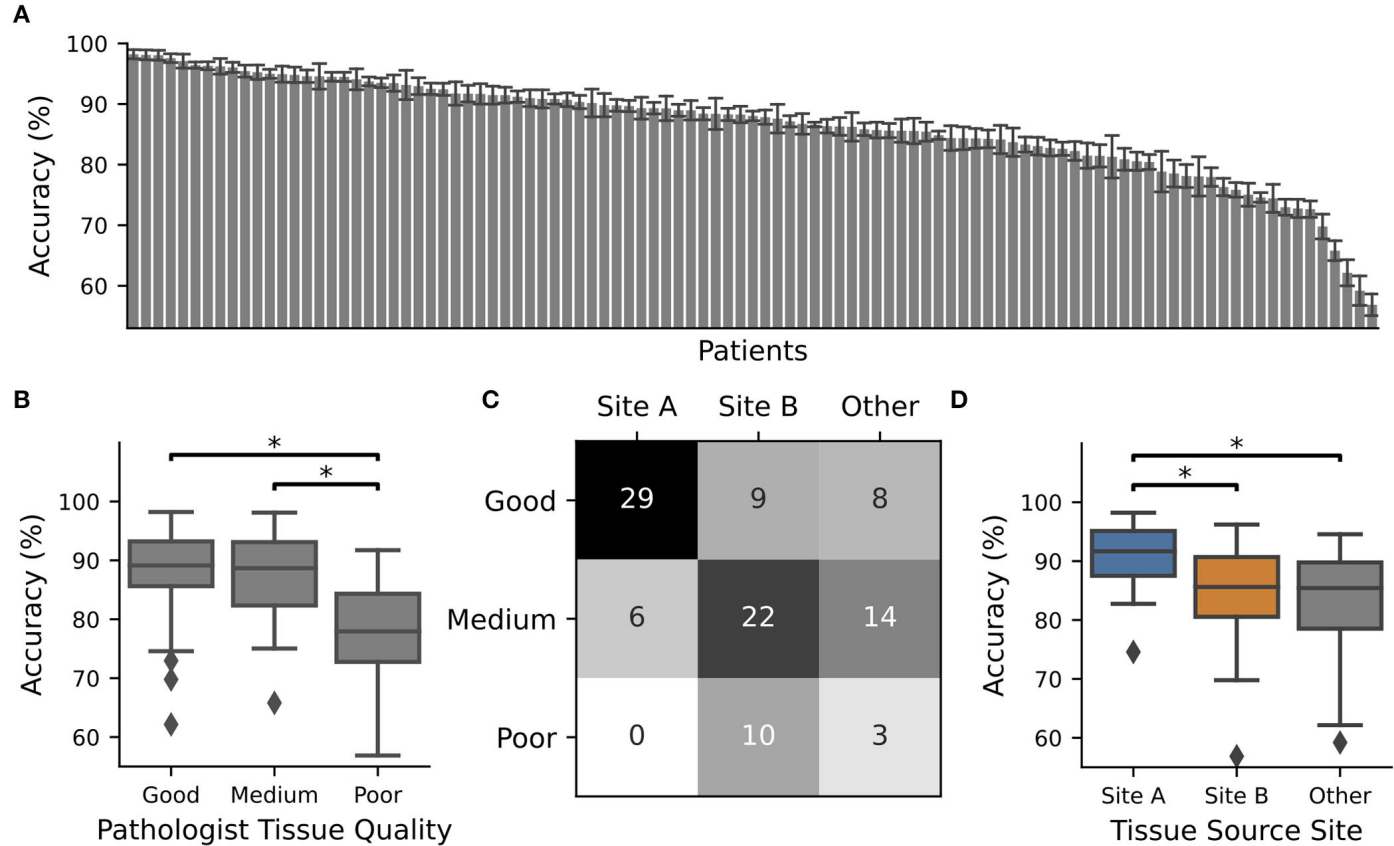
CNN performance depended on tissue quality and tissue source site. The TCGA-OV dataset was split into four subsets and 4 x 15 individual CNNs were trained with different train-validation splits using one subset as a test. The whole procedure was repeated 10 times. CNN performance was only recorded for WSIs in the test set. **(A)** Average accuracy for each WSI. The identifier with their accuracy is given in Supplementary Table S1. Error bars depict standard deviation. **(B)** Boxplot over the accuracy values of all slides with pathologist P1 assigned tissue quality. At least one category is significantly different according to an ANOVA (*p* = 0.00011), and significant differences between groups (*post-hoc t*-test with Bonferroni Holm *p*-value adjustments, *p* < 0.05) are marked with ^*^. **(C)** Contingency table for tissue source site vs. tissue quality. **(D)** Boxplot over the accuracy values of all slides from different tissue source sides. At least one category is significantly different according to an ANOVA (*p* = 0.00005) and significant differences between groups (*post-hoc t*-test with Bonferroni Holm *p*-value adjustments, *p* < 0.05) are marked with ^*^.

**Figure 2:**
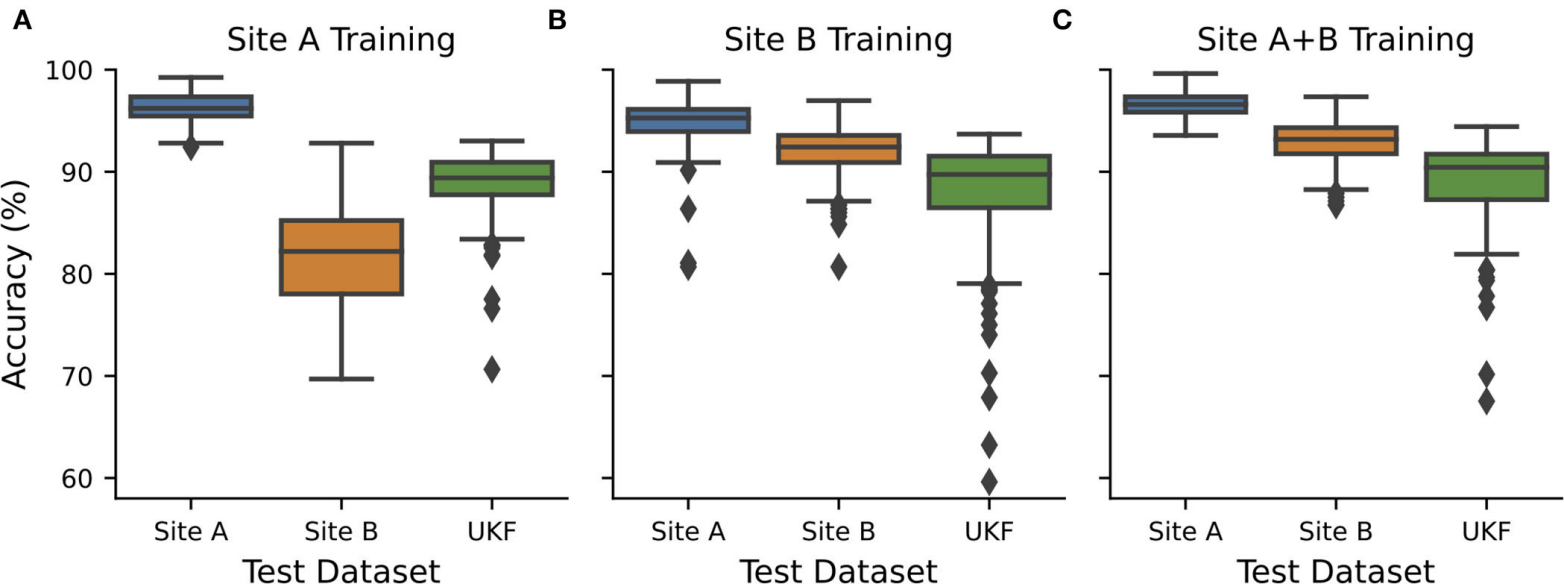
CNN transferability depended on the quality of training data. CNNs were exclusively trained and validated on data from **(A)** source site A (*n* = 21+7), **(B)** source site B (*n* = 27+7), or **(C)** both source sites (*n* = 48+14) and tested on hold-out test data from site A (*n* = 7) and site B (*n* = 7) and external data from UKF. Hold out test data were randomly chosen 10 times and every test 15 individual CNNs with different train-validation splits were calculated and the performance was measured. Boxplots show all recorded performances. Blue…site A; orange…site B; green…UKF.

**Figure 3:**
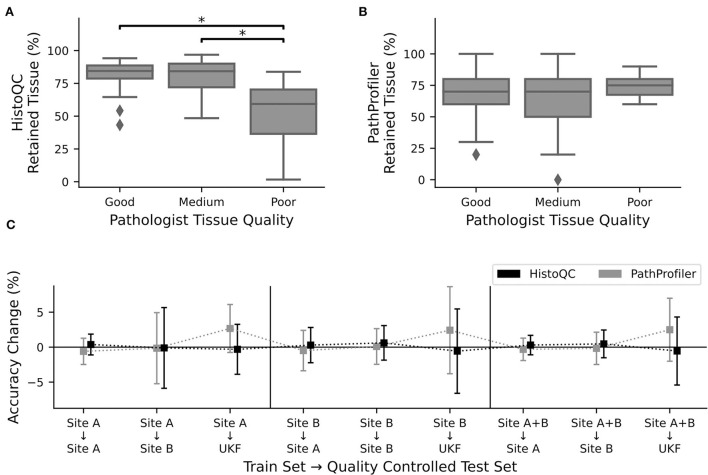
Quality control tools did not increase performance. Percentage of retained tissue per WSI by **(A)**. HistoQC and **(B)**. PathProfiler for the three different quality levels as assigned by pathologist one. For HistoQC, at least one group differs (ANOVA, *p*-value: 0.00003; *post-hoc t*-test with Bonferroni Holm *p*-value adjustments, *p* < 0.05 are marked with ^*^), and for PathProfiler, there are no significant differences between groups (ANOVA, *p*-value: 0.34). **(C)** CNNs trained for Figure 2 were used to calculate the performance on the test datasets, which were now quality controlled by either HistoQC (black) or PathProfiler (light gray) and do not contain tiles of low quality. Plotted is the performance difference between the original and quality-controlled test datasets. Error bars depict standard deviation.

**Figure 4:**
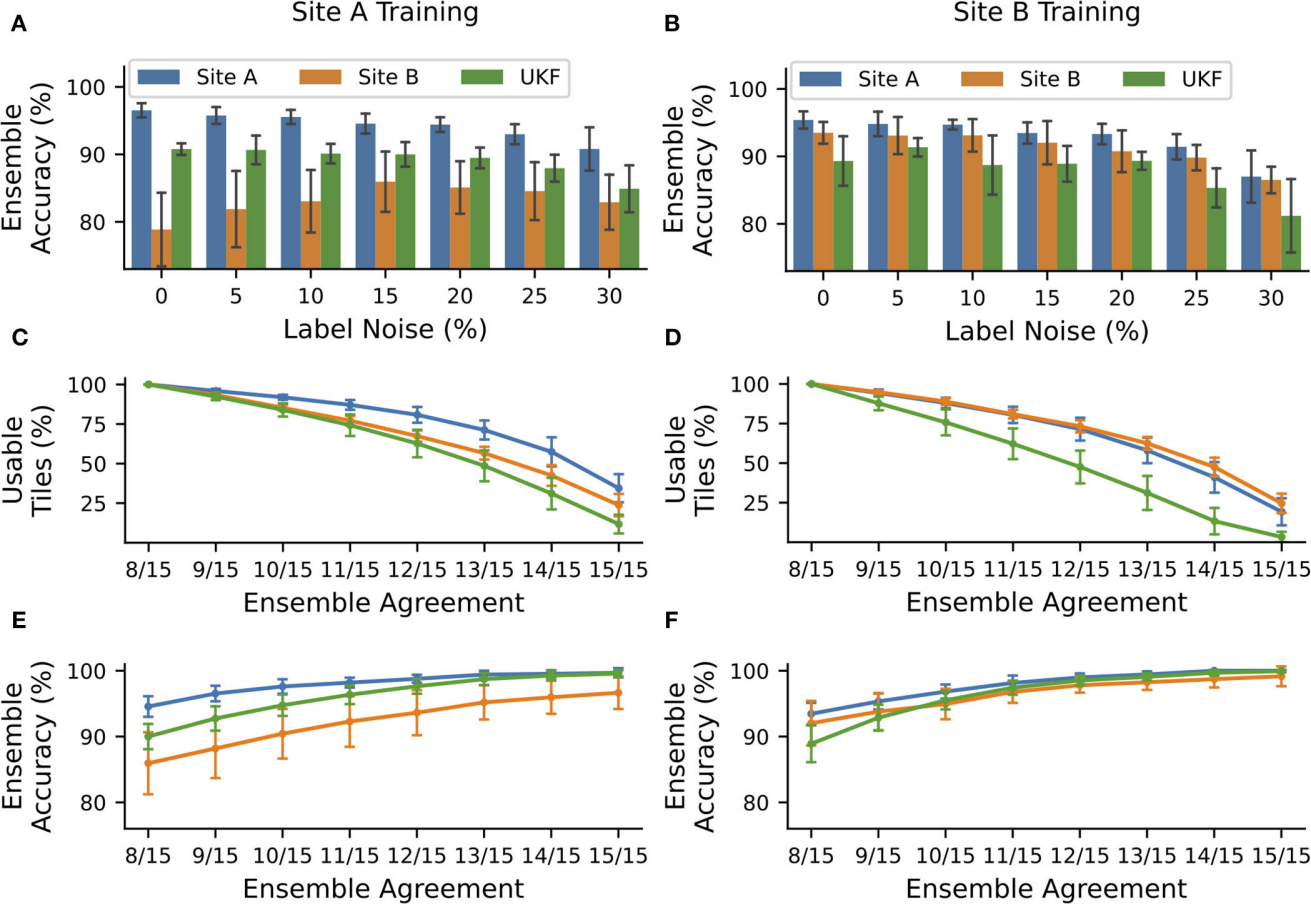
NoisyEnsembles could identify tiles with uncertain predictions. CNNs were trained with data from tissue source site A (left column) or tissue source site B (right column), while a defined amount of tiles was deliberately mislabeled in the training dataset. For each patient, either healthy or cancerous tiles were used during training. Hold-out test sets were chosen randomly and with the remaining data 15 CNNs with different train-validation splits were trained and combined with a bagging ensemble. The complete procedure was repeated 10 times. **(A,B)** Average ensemble accuracy for different test datasets. **(C–F)** NoisyEnsemble with a noise level of 15% during training. **(C,D)** Number of tiles with a valid ensemble prediction for the respective ensemble agreement. **(E,F)** Ensemble accuracy for individual levels of ensemble agreement. Predictions with smaller rates of the agreement were ignored in the respective categories. Error bars depict standard deviation.

Each CNN group/ensemble consisted of 15 CNNs with ResNet18 architecture. These ResNets were initialized with weights pretrained on ImageNet data. As an optimizer, we used Adamax as loss function the binary cross-entropy. Each CNN was trained for 10 epochs. During training, only the best iteration of a CNN based on validation accuracy was saved. The final performance of the group/ensemble was determined by averaging individual accuracies (group: Figures 1–3) or a majority vote by the CNNs (ensemble: Figure 4). For the noisy ensembles, we additionally repeated the training for seven different noise levels (0, 5, 10, 15, 20, 25, and 30%), with the corresponding randomly selected percentage of tiles having their labels flipped. All these procedures were repeated 10 times to obtain robust results. We defined our ensemble confidence by taking the amount of CNNs that matched the label predicted by the ensemble. All CNNs were created using Tensorflow version 2.4.1 (26). For each of the 10 training iterations, we created one train and test split, where the train split was additionally divided into 15 distinct train and validation splits for the individual CNNs of the group/ensemble. The splits occurred on a patient level ensuring that tiles from one patient were never part of multiple splits at the same time. For individual site training (Figure 2), test splits were created for the distinct tissue source site while keeping the test sets equal for Site A and Site B training. All performance values of the trained CNNs shown in the figures were obtained from test data only.

### Quality control tools

The quality control tools HistoQC (23) and PathProfiler (24) were used with default settings. Of the whole training dataset, HistoQC determined 2,843 tiles of the TCGA-OV dataset and 1,282 tiles of the UKF dataset to be used based on the mask_use. For PathProfiler, 2,692 tiles of the TCGA-OV dataset and 730 tiles of the UKF dataset were deemed usable if the corresponding area in the usblty_map had a value above 0.5.

### Statistics

For analysis of significance, we performed one-way ANOVA (f_oneway) as implemented in scipy.stats (27) for all groups, followed by a pairwise *post-hoc t*-test with holm *p*-value correction as implemented in scikit-*post-hocs*. We determined two group differences to be statistically significant if the pairwise *p*-value was below 0.05.

## Results

### CNN performance, tissue quality, and tissue source site are interconnected for the TCGA-OV dataset

To investigate the relationship between tissue quality and CNN performance, two pathology experts graded the histological images of the multicenter TCGA-OV dataset according to their quality into three quality categories: 1 (good), 2 (medium), and 3 (poor) based on criteria such as image sharpness, staining quality, tissue thickness, and tumor viability (Supplementary Figure S1). Most WSIs were classified as good (n_P1_ = 46; n_P2_ = 69), but there were also n_P1_ = 42/n_P2_ = 23 slides with medium overall quality and n_P1_ = 13/n_P2_ = 9 slides with poor overall quality. These numbers indicate that the quality assessment of the slides is naturally subject to a large inter-rater variability, however, the tendency is the same. In this case, expert two used systematically better scores, compared to expert one (Supplementary Figure S2). It is important to note here that although some WSIs were flagged as poor quality, all were still good enough for diagnostic purposes. Exemplary tiles extracted from the globally ranked WSIs are shown in Supplementary Figure S1. In addition, we also scored the tissue slides of an additional UKF dataset, which was newly generated at the institute for research purposes to ensure high quality. And indeed most slides were scored to have a high (n_P1_ = 21/ n_P2_ = 21) or medium (n_P1_ = 17/ n_P2_ = 21) quality and bad quality slides (n_P1_ = 3/ n_P2_ = 0) does hardly occur.

Next, we trained individual CNNs to separate cancerous from non-cancerous regions using 75% of the TCGA-OV dataset for training and validation and 25% for testing. We repeated the data splits so that every patient was 10 times part of the test set. The average performance of the individual CNNs was only recorded while patients are located in the test dataset. We observed that for approximately one-third of all patients, the performance was high (accuracy > 90% for *n* = 38 patients). However, there were also patients with low performance, which was going down to less than ~60% (Figure 1A) and is close to random guessing.

In general, the patients with the best performance were those who had good tissue quality as assessed by expert one, while those with the poorest performance tended to have low quality scores. There was a significant difference in performance between tissue sections of good and poor quality (Figure 1B). This observation did not depend on the expert who evaluated the tissue quality as the same tendency was present when the slides were scored by expert two, or if only slides were used where both experts agree (Supplementary Figure S3). In addition, we found that tissue quality corresponds with different tissue source sites (Figure 1C) and therefore performance was also related to this characteristic (Figure 1D).

### Tissue source sites influence CNN transferability

We now wanted to investigate how these intrinsic quality differences in the WSIs influence the transferability and performance of trained CNNs. Therefore, we trained CNNs exclusively on data obtained from one tissue source site. The different tissue source sites could be also identified to a certain degree using CohortFinder (28), which is based on HistoQC (23) (Supplementary Figure S4). Training and testing on tissue from site A, with high quality as assessed by the pathologist, results in high accuracies with a median of 96.2% (Figure 2A, AUC: 0.98). However, using the tissue with low quality from site B for testing, a strong decrease in the accuracy to 82.2% (AUC: 0.86) was observed. Testing with an external dataset from UKF obtained again an intermediate accuracy of 89.4% (AUC: 0.93).

When the network was trained with data from source site B, which tend to have a low quality, surprisingly, this time the images from source site A performed better compared to those from site B as well (median accuracy/AUC site A:95.3%/0.97 vs. site B:92.4%/0.95; Figure 2B). In addition, the performance for slides of lower quality increased as the network has already seen comparable ones during training. The performance on the external UKF data is comparable for the CNN trained with low-quality data (median accuracy/AUC: 89.7%/0.94) compared to the CNN trained with higher quality. Using data from both source sites for training (so also more data are available for training), we could slightly improve the accuracies (Figure 2C, median accuracy/AUC site A:96.6%/0.98 vs. site B:93.2%/0.96), indicating a small beneficial influence of the addition of the high-quality slides of site A.

To further strengthen our hypothesis that tissue quality is indeed one of the main factors influencing the performance of CNNs and to rule out unexpected interactions between datasets of different origins, we also checked performance only within the data of site B in dependence on the quality (Supplementary Figure S5), as site B is the only tissue source site containing all three quality levels. Here, we found that the CNNs always performed better on the WSI's with a good and medium tissue quality than those with low quality. In addition, it is important to note that the highest accuracies could be achieved by using medium-quality slides during training, again highlighting their importance for training high-performing CNNs.

To ensure that the observed effects were not caused by different color appearances, we repeated the experiments with stain-normalized (19, 20) tiles. The observed accuracies were higher, and especially the difference between site B and the UKF data nearly vanished. However, the overall pattern remained the same: Models trained with high-quality data were poorly transferable to low-quality data, and models trained with medium- to poor-quality data performed better on high-quality data (Supplementary Figure S6).

Taken together, these results indicate that it is beneficial to include images of a lower quality during training to obtain the best performance and transferability in the end. Nevertheless, once these algorithms are used in a routine setting, slides with low quality should be avoided if possible to obtain the best possible performance and to avoid false predictions.

### Tissue quality control tools do not increase performance

After recognizing how crucial the influence of the used tissue was, we asked the question of whether we could further improve our prediction results by the use of the automated quality control tools, PathProfiler (24) and HistoQC (23). As HistoQC does not provide a slide-wide score, we had a closer look at the amount of tissue, which is recommended to use. Here, we saw that the percentage of usable tissues was significantly higher for the slides with a good (1) and medium (2) quality compared to the slides of poor (3) quality as assessed by expert one (Figure 3A). In opposite, for PathProfiler, we did not see any differences between the different quality stages (Figure 3B); however, it is important to mention that PathProfiler is not yet tested for non-prostate tissue. Again these observations did not depend on the expert who scored the slides for quality (Supplementary Figure S7).

Following our idea that poor-quality slides should be avoided during the application of CNNs, we cleaned up our test datasets and removed all image patches which were rejected by HistoQC or PathProfiler. Surprisingly, instead of the expected increase in the accuracy values, we did not observe any change for the images of sites A and B, irrespective of the data used during training (Figure 3C). Only for the external UKF data, there was a slight increase in accuracy after the usage of PathProfiler for some training and validation splits (Figure 3C). Also, after cleaning up the training and validation datasets, no improvements in the accuracies were observed for one of the datasets. If there is an effect at all, the accuracy is reduced (Supplementary Figure S8).

To better understand why there was no change in performance, we had a closer look at the excluded image patches (Supplementary Figure S9). The tiles removed by HistoQC were partially meaningful, as a pathologist would approximately remove 3/5 tiles as, for example, no vital tissue was present or the contrast was too low. Nevertheless, we furthermore recognized that the border regions of a tissue section were excluded because of blurriness, which could not be validated by a pathologist. But image artifacts such as, for example, the border of air bubbles or dust in the background were reliably detected by HistoQC (Supplementary Figure S10). In contrast, for PathProfiler, most of the tiles were fine for a pathologist, this fits the nonexisting difference in the number of removed tiles for different quality levels (Figure 3B).

Although the HistoQC results were similar to the pathologists' assessment of tissue quality, the exclusion of tiles that should be of poor quality according to HistoQC and/or PathProfiler did not improve the performance of our CNNs. Therefore, we decided to use the full datasets.

### NoisyEnsembles can improve CNN transferability to data with lower quality

To now find an alternative way to decide which image patches are suited as input for a trained deep learning approach and to recognize if a prediction is uncertain, we trained bagging ensemble CNNs and had a closer look at the ensemble confidence (number of CNNs having the same prediction). Recently, we have shown that using only securely predicted image tiles can end up in low error rates (6). To avoid the problem of overconfident ensemble predictions, we inserted some label noise into the training dataset. Therefore, we only used cancerous or healthy tiles of each patient and then swapped the label of a certain amount of tiles. As expected, the accuracy of the individual CNN was decreasing with an increasing amount of noise in the training data (Supplementary Figure S11). But the insertion of noise had the desired effect: With an increasing amount of noise, the number of CNNs with the same predictions was decreasing. And importantly, the concordance was always higher for correctly predicted tiles compared to false predictions (Supplementary Figure S12).

The overall accuracy of the ensemble predictions stayed nearly constant (change under 2%) for small noise levels ( ≤ 15%) when trained with high-quality data coming from tissue source site A and tested on data of the same site or the UKF. Even more importantly, the accuracy for WSI's with lower quality coming from tissue source site B increased by ~7% for these noise levels, and thus the ensemble was getting more robust toward the images with the lower quality (Figure 4A). Also for ensembles trained with data from site B, there was no strong decrease (under 2%) in performance for small levels of label noise in the training datasets (Figure 4B).

As mentioned before, for each patient, we either used healthy or cancerous image tiles. This is an essential characteristic of our NoisyEnsemble: If both classes of one patient are used simultaneously, the desired effect of increased performance for unseen low-quality data did not occur (Supplementary Figure S13).

Also, the amount of data used during the training of course influences CNN performance. Ensembles trained on site A data without noise and tested on site A and UKF data get, as expected, better with higher amounts of data. Interestingly, the performance of the non-noisy ensembles also increased with higher amount of training data for the low-quality site B test data, but they perform worse if trained on the entire dataset, suggesting that the non-noisy models were overfitted for the high-quality data (Supplementary Figure S14). In contrast, such a decrease in performance was not observed for our NoisyEnsemble and the ensembles with noise always outperformed the non-noisy variant for low quality data (Supplementary Figure S14). In addition, the performance gap between the NoisyEnsemble and the non-noisy version, which was observed for the high-quality datasets was getting smaller with higher amounts of data (Supplementary Figure S14).

### Images with unsecured predictions during testing could be identified by NoisyEnsembles

Next, we wanted to exclude images from the NoisyEnsemble (noise level: 15%), which were likely to have a wrong prediction. For this reason, we determined the ensemble agreement and considered tiles unclassifiable if the agreement was below a certain threshold. When we applied a threshold of 100% agreement (15/15 CNNs with identical prediction), over 50% of all tiles could not be classified any longer. However, it is important to mention that the number of removed tiles was always the smallest for the site which was used during training. This indicates that the ensemble was used to this kind of tiles, while from the external UKF dataset, always the highest number of tiles was removed (Figures 4C,D). With increasing ensemble agreement, the accuracy for the remaining tiles increased for all test datasets, no matter which dataset was used during training (Figures 4E,F). In addition, the accuracy for the external UKF dataset even increased to the same level as the source site used during training. Also, the performance gap, that occurred when CNNs were trained with high-quality data (Figures 2A, 4A), between the high-quality test images from site A and low-quality test images from site B was narrowed (Figure 4C). With the 100% agreement, we nearly achieved an accuracy of ~100% for most of the test datasets, irrespectively of the used training data. The number of false predictions (FP and FN) decreased with increasing ensemble agreement and if only a small ensemble agreement was required, more false than correct predictions were discarded (Supplementary Figure S15).

A visual inspection by a pathologist of tiles from site B removed by the NoisyEnsemble trained on site A data again revealed a connection to the tissue quality: Removed tiles, for example, mainly have low contrast, which is especially true for confidence levels of 9/15 to 13/10 (Supplementary Figure S16). To now understand the paradox that both HistoQC and the NoisyEnsemble removed tiles related to tissue quality but only one of them resulted in a performance increase, we had a closer look at the overlap of the removed tiles (Supplementary Figure S17). Here, we realized that the ensemble removed fewer tiles at high agreement levels (12/15), and also observed that the overlap was always smaller than the number of tiles removed by one method. Even for the highest agreement level (15/15), HistoQC removed tiles that were retained by the ensemble and missed to exclude the relevant tiles to end up with the highest possible performance (Supplementary Figure S17).

To sum up, the use of NoisyEsembles (trained with the introduction of label noise) can improve the transferability of CNNs to lower quality data, however, it could not increase the performance for data with the better or same quality. The additional usage of ensemble confidence is suited to increase performance further and mainly removes wrongly predicted tiles of low quality.

## Discussion

### Datasets for training and application of CNNs

Tissue slide quality and the presence of artifacts are important in computational pathology and it is necessary to understand the interrelationship of the chosen material and the performance of trained deep learning algorithms. Here, we provided some insights and showed that there are unexpected large performance differences between individual WSI's (all suited for diagnostics) coming from the same TCGA-OV dataset, but from different tissue source sites (Figure 1). These WSI's differ in terms of their tissue quality (Figure 1, Supplementary Table S1). In general, differences based on first- and second-order image characteristics between WSI's of different source sites within TCGA datasets were described earlier (29), and therefore it was shown that it is important not to mix sites for training and model evaluation to not overestimate CNN performance. In addition, we now showed that tissue quality is an important factor that determines the performance of CNNs: High-quality WSIs always perform well, irrespectively of the used training data (Figure 2). In general, it is recommended to use different tissue source sites to increase image variability during training to improve the generalization of CNN models (30), which is also in line with our findings as the model with both source sites performed best in our hands (Figure 2). In addition, we showed that the tissue quality is an important driver of that observation. Furthermore, our study pointed out that it is beneficial to include medium- and low-quality slides in training to achieve the desired transferability—including only multiple sites with the highest tissue quality will most likely not achieve the desired effect. Next to the ideal composition of the training data, we also introduced NoisyEnsembles (Figure 4), which improved the transferability to datasets with other properties by selectively inserting label noise during training. Like other ensemble methods (6, 31), our NoisyEnsembles also have the potential to increase the overall performance of the deep learning application (Figures 4A,B vs. Supplementary Figure S11). For testing, it is recommended to sample the possible image space as well as possible (11). However, during the application of CNNs in a routine setting, we recommend paying attention to the best possible quality, as the performance was directly linked to the tissue quality (Figure 2, Supplementary Figure S1). Our NoisyEnsemble is also able to discard or mark images that have bad quality and are likely to contain erroneous predictions (Figure 4, Supplementary Figure S16), a feature that could help to improve clinicians' trust in the technology. In addition to this already implemented feature, explainable AI (XAI) methods such as Grad-CAM (32) or LRP (33) could further help to overcome the resilience in clinics in future. First methods also combine ensemble predictions with XAI and, for example, provide a weighted combination of the single CAMs (34) to visualize which image regions are important for the actual decision. Such techniques could of course also be applied to our NoisyEnsembles: By calculating the average Grad-CAM of an ensemble, the algorithm's decisions can be visualized and validated. A physician can assess whether the prediction fits the areas considered by the algorithm; this is especially helpful for images with uncertain predictions (Supplementary Figure S18).

### Label noise and CNN training

In general, label noise is an undesired phenomenon, as it usually decreases the performance of deep learning algorithms (35), which we also observed during the training of the individual CNNs (Supplementary Figure S11). So, most methods try to find ways to overcome the problem of label noise (35). However, we did it the other way around and inserted the label noise intentionally, as it is also known that label noise can introduce penalty terms, similar to the regularization approaches, which have the potential to improve generalization (36). By the use of the ensemble, we could nearly completely overcome the negative effect of noise on the same data quality and, indeed, we were able to improve the transferability to unseen, lower quality data (Figure 4). Taking all test sets together, the overall performance could be even improved by the insertion of noise (Supplementary Figure S19), and the slight decrease in performance for high qualities of data vanished if only secure predictions are taken into (Supplementary Figure S20), however, in this case, a prediction is not possible for all tiles.

So, a frequently used approach to tackle label noise is to relabel, remove, or re-weight falsely labeled data points from the training dataset (35), which could be also done with the help of ensembles (37). But these classical methods cannot be applied to NoisyEnsembles, because then the positive effect of label noise on transferability would be lost. However, there are also alternative approaches that can overcome label noise by, for example, using evidence-based accumulation clustering which processes several samples in a batch and correct the individual predictions based on the observed nearest neighbors in the different classifiers (38). In future, it is interesting to test if such methods, not dealing directly with the label noise, could keep the positive effect on the transferability to unseen data while improving the performance of data, which is similar to training data.

### Further intrinsic differences between the datasets of sites A and B

In general, there are different possible options why datasets collected at different tissue source sites behave differently in training and testing. First, it is important to mention that next to the different overall quality (Figure 1), there are also some additional intrinsic differences between the patients of the different tissue source sites in the TCGA-OV dataset: Patients from site A live significantly longer (overall survival, disease-specific survival, and regression-free survival) compared to patients from site B. And in addition, patients from site A are significantly younger at the timepoint of diagnosis (Supplementary Figure S21). Principally that connection between age and survival is well-known in ovarian cancer (39). But it is also possible that the age of the patient also influences the quality of the tissue itself and so the performance of the deep learning algorithm decreases: For older patients, it is known that the composition of the ovarian extracellular matrix (e.g., increase of collagen and decrease of elastin) changes with age (40), and so the cutting properties of the tissue is altered. Which in turn could lead to unevenly distributed tissue thicknesses or different amounts of cracks. Moreover, different fixation and staining protocols or laboratory equipments could be responsible for our observation of varying tissue quality.

A second intrinsic difference between the images of tissue source sites A and B is different JPEG compression levels (site A: quality 30; site B: quality 70). It is well-known that very strong JPEG compression influences CNN performance negatively (12–14). However, the results obtained here were the other way round: The more strongly compressed images from site A perform better compared to the images of site B indicating that other factors are more important. It is also important to note that severe effects of JPEG compression only begin at a compression level of 85% (14), which was not used for any of the WSI's of the TCGA-OV dataset.

In addition, it is of course also possible that one center (e.g., based on its physical location or its role as a reference center) simply has more clinically advanced cases. And indeed, a pathologist scored more cases as challenging for site B compared to site A and CNNs of course performed slightly better on slides that were evaluated by a pathologist to be clear in distinguishing cancer and non-cancerous regions (Supplementary Figure S22A). But importantly, within one quality level or one source site, there are no significant differences between the differently ranked slides (Supplementary Figures S22B,C), and so we conclude that an uneven distribution of difficult cases is not responsible for the effects observed in here and it is indeed the tissue quality which makes the difference regarding CNN performance and transferability.

## Conclusion

The transferability of CNNs is affected by the data quality during training, and it is beneficial to include mainly medium- and low-quality data in the training set. CNNs achieve the best test results with high data quality, regardless of the quality of the data with which they were trained. Therefore, care should be taken to ensure the highest possible data quality when using them later. Since it is often difficult to estimate how different the data used later will be, it is still important to come up with methods that can deal with this problem: Our NoisyEnsembles increase the transferability to lower quality data and additionally discard inappropriate data points to avoid incorrect predictions in routine clinical use. To enable this, the NoisyEnsemble was trained with ~15% of wrong labels to enforce a greater variability within the ensemble predictions and thus become more robust against image diversity.

## Data availability statement

The datasets presented in this article are not readily available because WSI's are patient data and could not be made publicly available due to legal restrictions. Source code will be made available after publication. Requests to access the datasets should be directed to nadine.flinner@kgu.de.

## Ethics statement

Written informed consent was obtained from the individual(s) for the publication of any potentially identifiable images or data included in this article.

## Author contributions

NF conceived and designed the study. RM performed the experiments and analyzed the data. LH performed the normalization of the staining. KB participated in the collection of tissue samples. SG, PZ, and PW provided their histopathological expertise. RM and NF wrote the first draft of the manuscript. All authors contributed to the interpretation of the results, critically revised the manuscript, and approved the final version of the manuscript.

## Funding

RM and NF were supported by the Mildred Scheel Career Center (MSNZ) Frankfurt, and LH was supported by the Alfons and Gertrud Kassel Foundation.

## Conflict of interest

Author PW has received consulting fees and honoraria direct/institutional for lectures from Bayer, Janssen-Cilag, Novartis, Roche, MSD, Astellas Pharma, Bristol-Myers Squibb, Hedera Dx, Thermo Fisher Scientific, Molecular Health, Sophia Genetics, Qiagen, Eli Lilly, Myriad, and Astra Zeneca. He has received research funding from Astra Zeneca and Thermo Fisher. The remaining authors declare that the research was conducted in the absence of any commercial or financial relationships that could be construed as a potential conflict of interest.

## Publisher's note

All claims expressed in this article are solely those of the authors and do not necessarily represent those of their affiliated organizations, or those of the publisher, the editors and the reviewers. Any product that may be evaluated in this article, or claim that may be made by its manufacturer, is not guaranteed or endorsed by the publisher.
