# Supplementary material for: How to learn with intentional mistakes: NoisyEnsembles to overcome poor tissue quality for deep learning in computational pathology
Source: Front Med (Lausanne). 2022 Aug 29;9:959068. doi: 10.3389/fmed.2022.959068 (PMC9464871; doi:10.3389/fmed.2022.959068)
Supplement: Supplementary file 1 [file Data_Sheet_1.pdf]

Supplementary material for

NoisyEsembles: to overcome low tissue quality in computational pathology

Robin S. Mayer*, Steffen Gretser*, Lara E. Heckmann, Paul K. Ziegler, Britta Walter, Henning Reis, Katrin Bankov, Sven Becker, Jochen Triesch, Peter J. Wild, Nadine Flinner

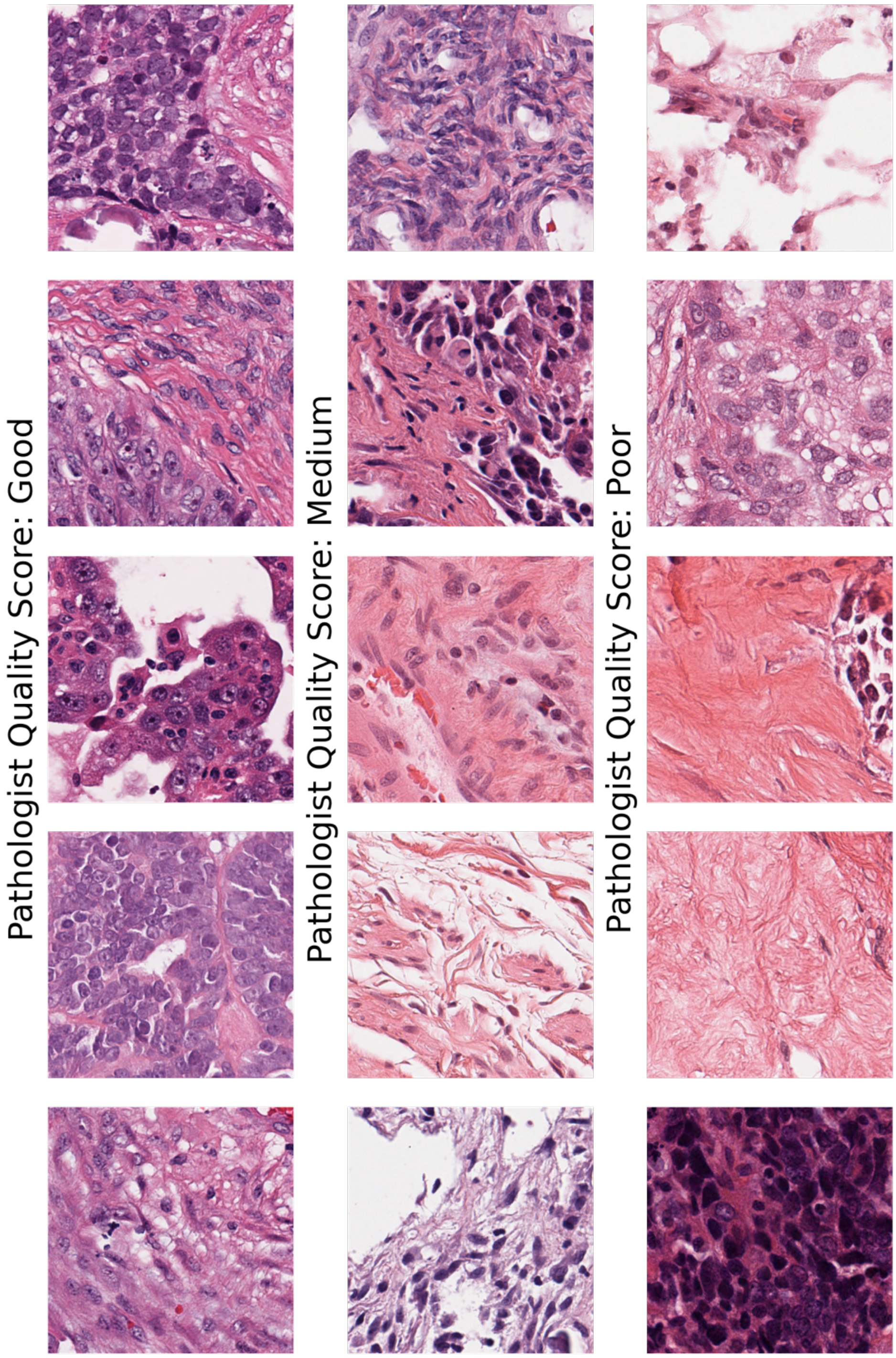

Supp Figure 1. Tiles with good, medium and poor quality. For each category, 5 tiles from WSI's with a globally assigned good, medium or poor tissue quality by a pathologist were chosen randomly for visualization.

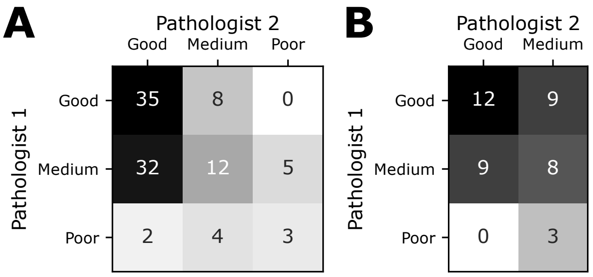

Supp Figure 2. Inter rater variability for tissue quality. Contingence table for two different raters (P1 and P2) evaluating the tissue quality of **(A)** the TCGA-OV dataset and **(B)** the UKF dataset, where slides were generated specifically for research purposes.

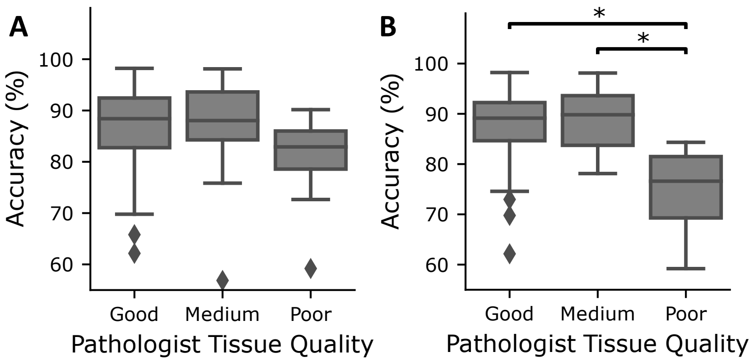

Supp Figure 3. CNN Performance depend on Tissue Quality, independently of the rator. Boxplot over the accuracy values of **(A)** all slides where pathologist P2 assigned tissue quality and **(B)** slides where both experts assigned the same quality level. No significant differences were observed in (A) with an ANOVA (p=0.090). At least one category is significantly different in (B) according to an ANOVA (p=0.008), significant differences between groups (pos-hoc t-test with Bonferroni Holm p-value adjustments, p-value<0.05) are marked with *.

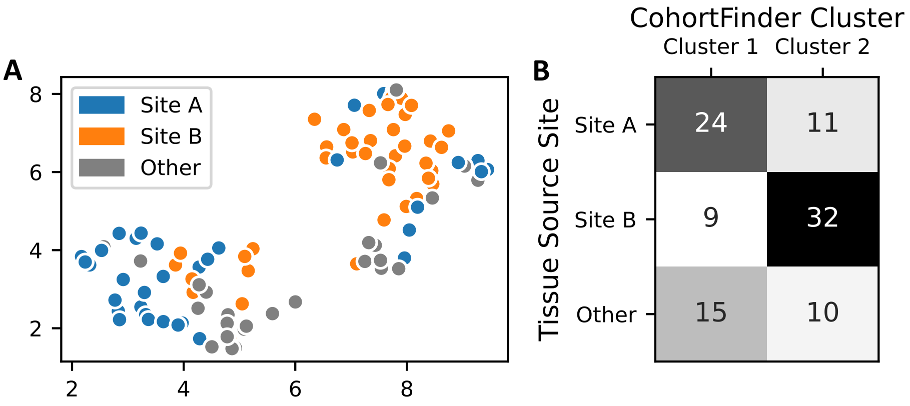

Supp Figure 4. Cohort Finder Results. (A) In the embedding plot two different subclasses appear. Samples are colored by tissue source site. Most of the site A patients are located in the lower cluster (C1) and most of the patients from source site B are located in the upper cluster (C2). The images from other source sites are distributed over both clusters. **(B)** Confusion matrix comparing the tissue source site with the corresponding clusters as identified in CohortFinder.

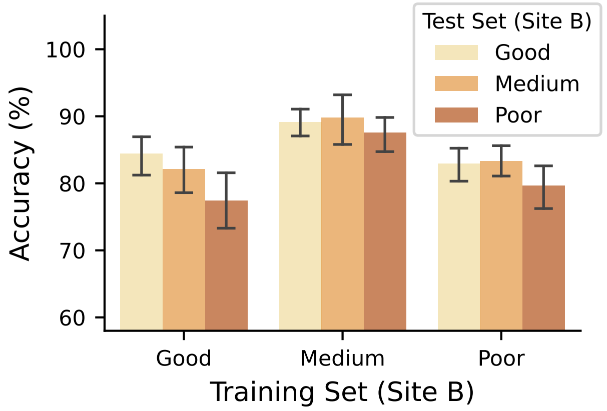

Supp Figure 5. Influence of Tissue Quality within the site B dataset. CNNS were trained and validated on data from site B (n=5+1 patients) and tested on hold-out test data from site B (n=3 patients). Here, the different quality levels were treated independently.

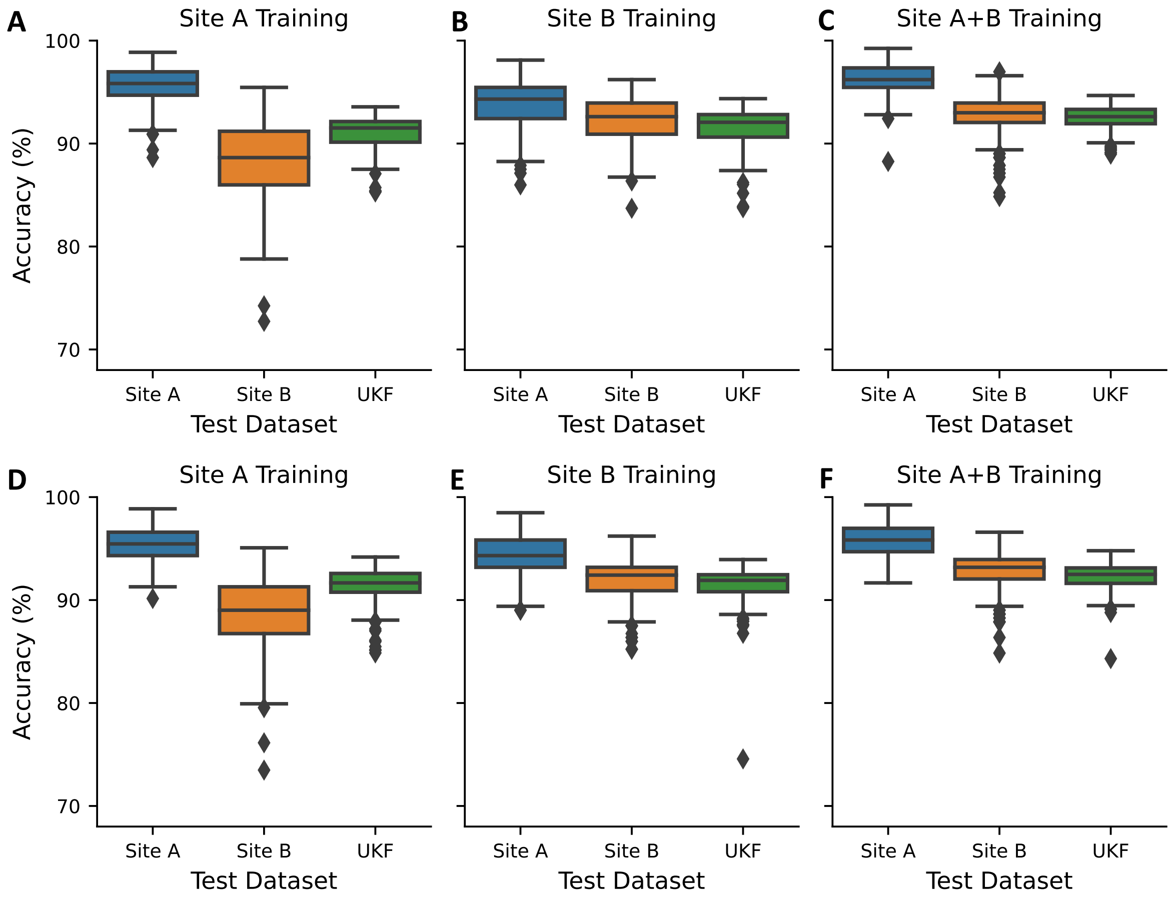

Supp Figure 6. Influence of Stain Normalization. Experiments as described in Figure 2 were repeated in the same manner as described there, however this time all images from the training, validation and test datasets were normalized using the **A.** Macenko or **B**. Vahadane stain normalization method.

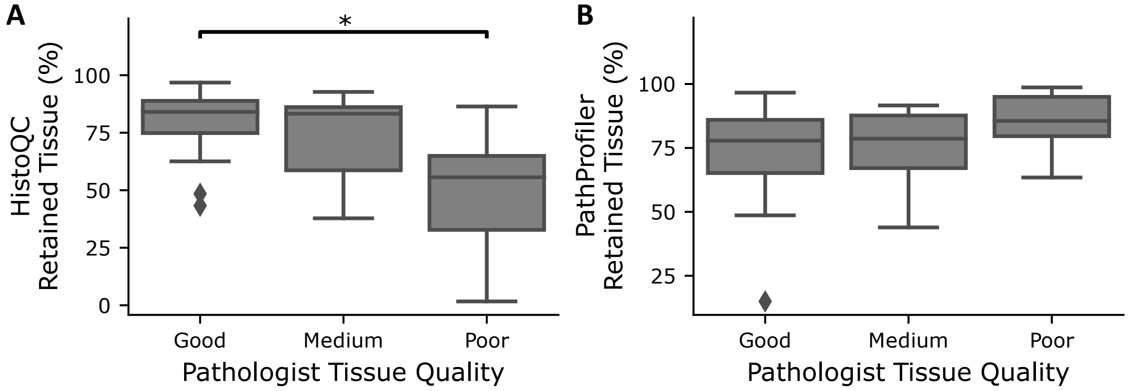

Supp Figure 7. Tissue retained by quality control tools in relation to tissue quality. Percentage of removed tissue per WSI by **A.** HistoQC and **B.** PathProfiler for the three different quality levels as assigned by pathologist P2. For HistoQC at least one group differs (ANOVA, p-value: 0.00003; pos-hoc t-test with Bonferroni Holm p-value adjustments, p-value<0.05 are marked with *), for PathProfiler there are no significant differences between groups (ANOVA, p-value: 0.53).

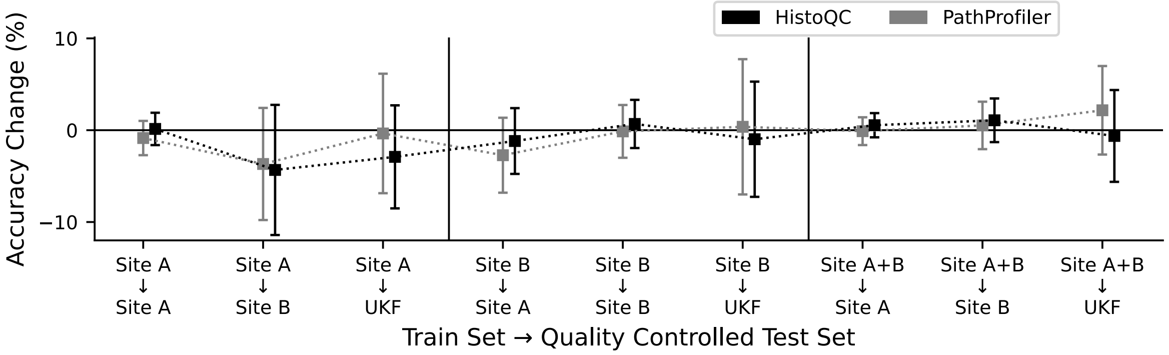

Supp Figure 8. Application of Quality Control tools during Training and Testing. Experiments as described in Figure 3 were repeated in the same manner as described there, however this time all tiles (so training, validation and test datasets) were cleaned with one of the quality control tools.

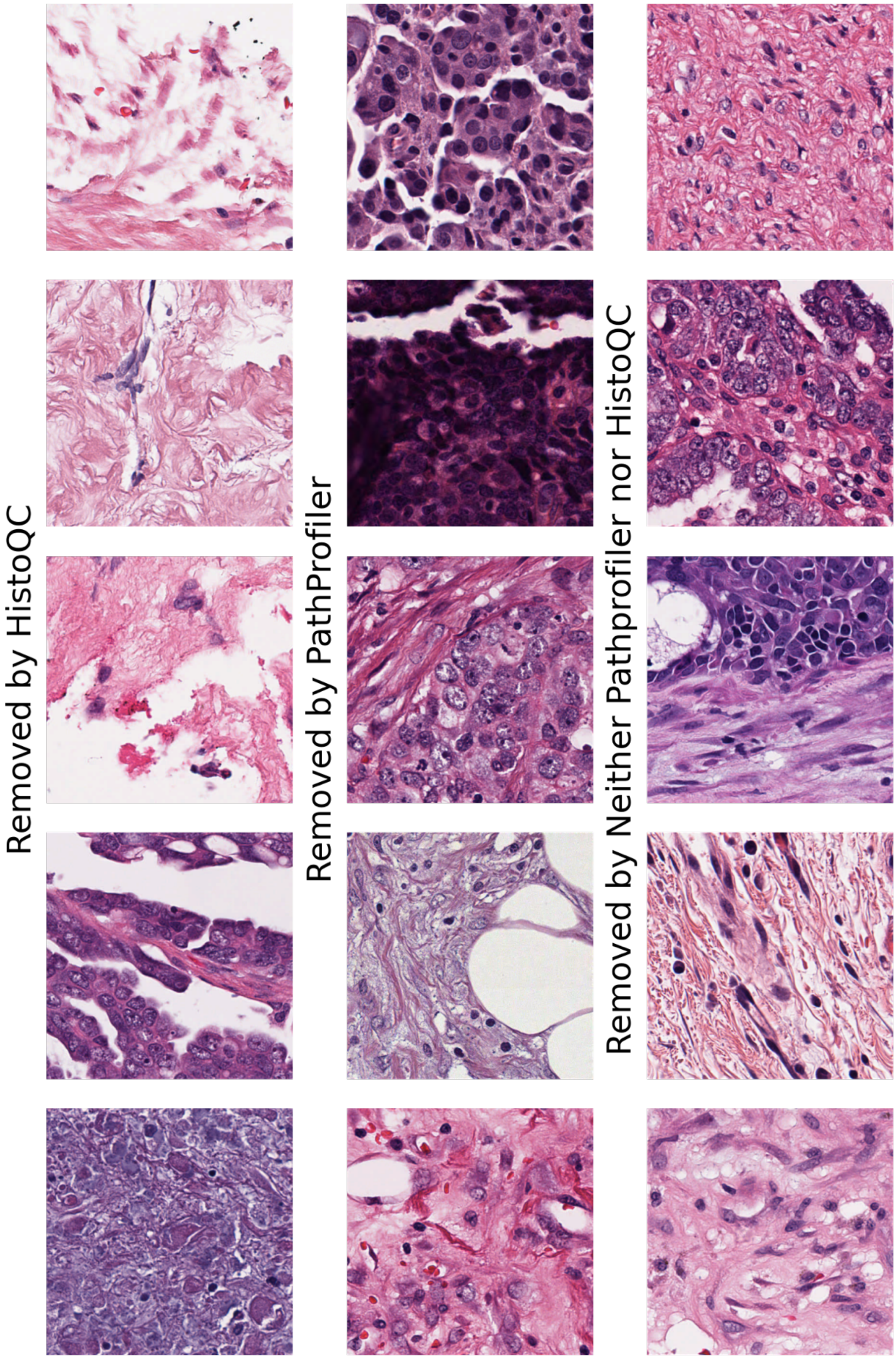

Supp Figure 9. Tiles excluded by quality control tools. For each category, 5 tiles which were removed by HistoQC, PathProfiler or which were kept by both were chosen randomly for visualization.

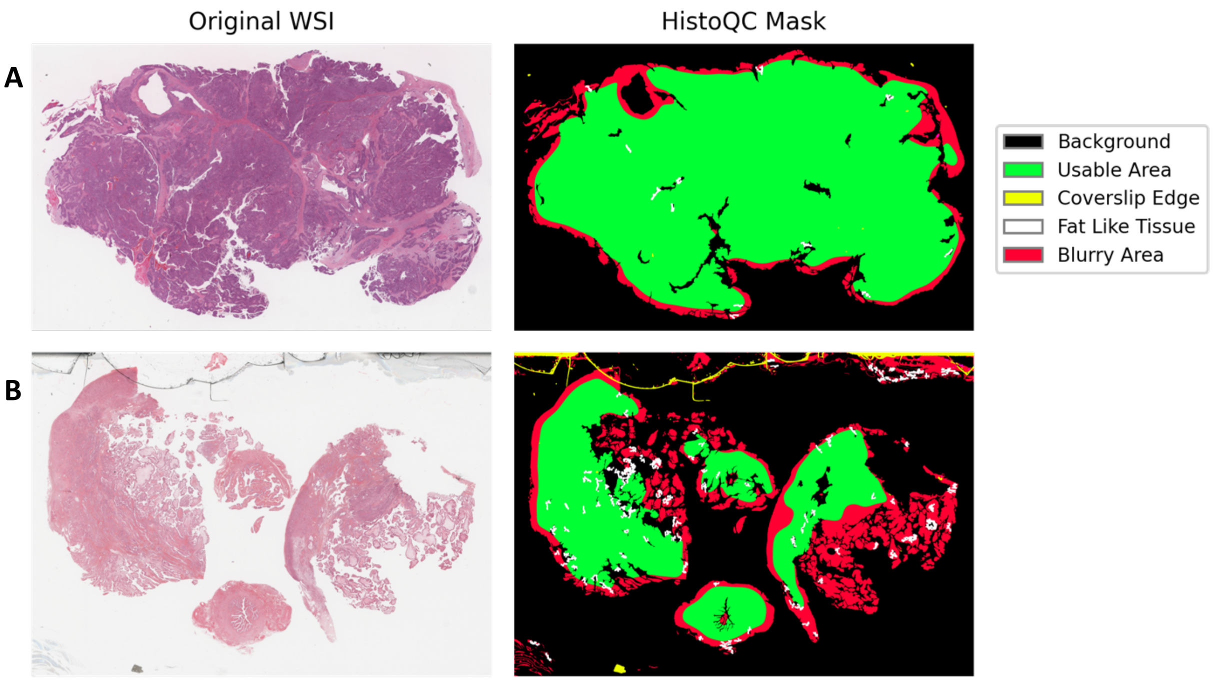

Supp Figure 10. HistoQC Masks. Visualization of WSI images together with the HistoQC results. **A.** TCGA-23-1021 **B.** TCGA-25-1321

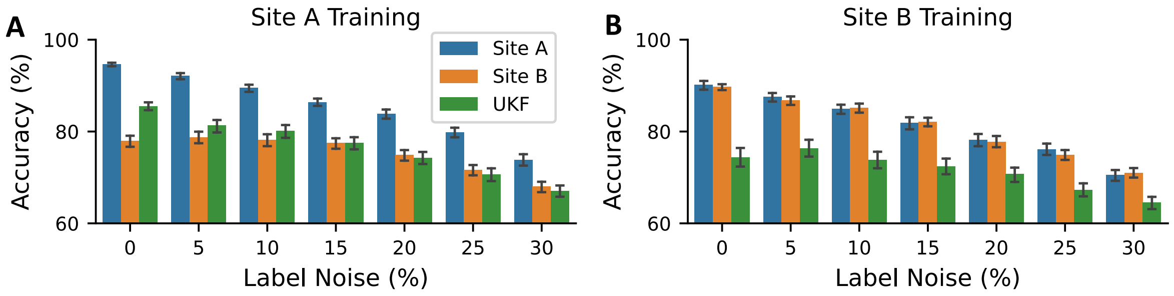

Supp Figure 11. Performance of individual cNNs in dependance of label noise during training. CNNs were trained with data from **A.** source site A and **B.** source site B. Test datasets are indicated by color: blue…site A; orange…site B; green…UKF.

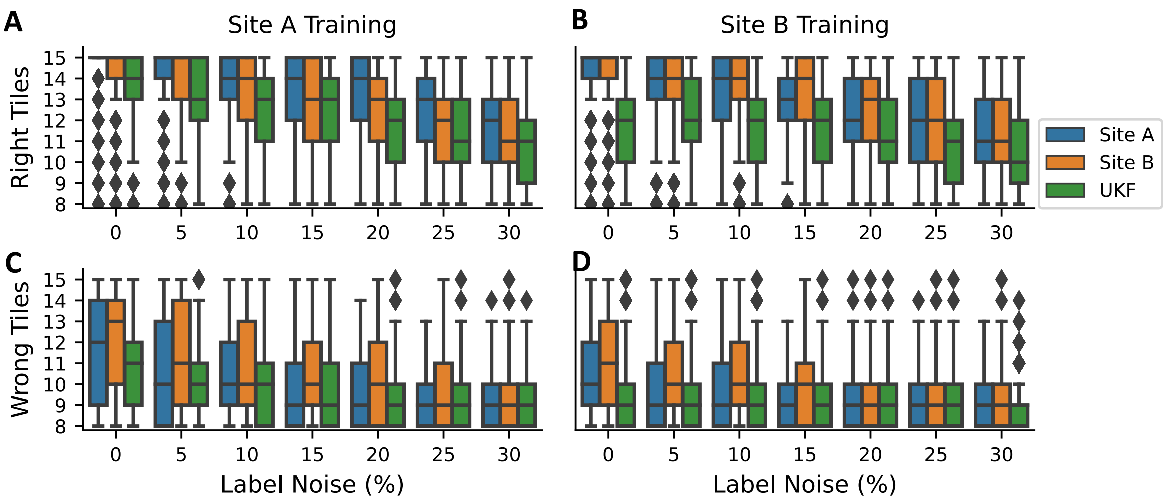

Supp Figure 12. Ensemble Agreement for correct and incorrect predictions. NoisyEnsembles were trained with data from **A+C.** source site A or with data from **B+D.** source site B. Test datasets are indicated by color: blue…site A; orange…site B; green…UKF. The boxplots shown the ensemble agreement for **A+B.** correctly and **C+D.** wrong predicted tiles in dependence of the used label noise.

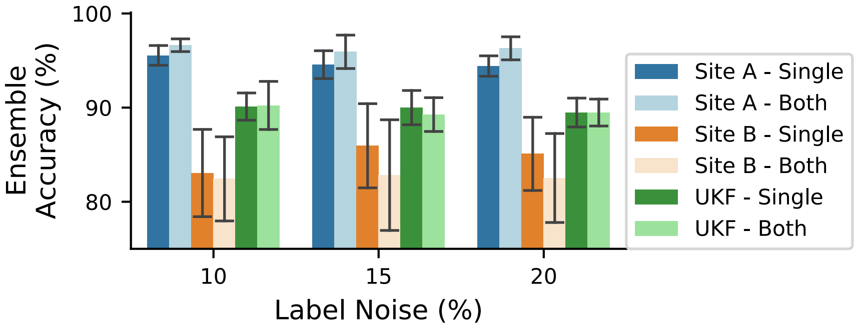

Supp Figure 13. Noisy Ensembles with symmetric noise. We repeated our experiments for the NoisyEnsambles, using this time both classes (cancerous and healthy) for every patient and also inserted the noise symmetrically on both classes (light colors). Results wehre a single class is used for training are shown in dark color for comparison. This time the performance for the unseen images of site B keeps constant (light colors) and is worse compared to the performance of the NoisyEnsemble, which is only trained with one class per patient: NoisyEnsemble $_{\text{both labels; 15\% noise}}$: 82.83±9.20; NoisyEnsemble $_{\text{one labels; 15\% noise}}$: 85.95±4.71;

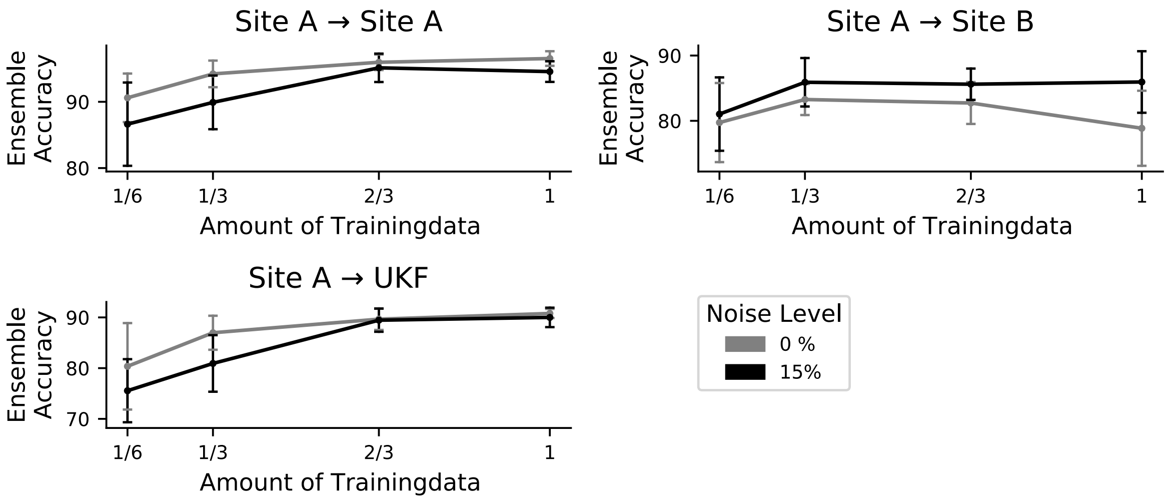

Supp Figure 14. Noisy Ensembles with different amounts of data. Ensembles (noise level: 0% and 15% were trained with different amounts of the complete high quality site A dataset and tested on **(A)** site A data **(B)** site B data and **(C)** UKF data.

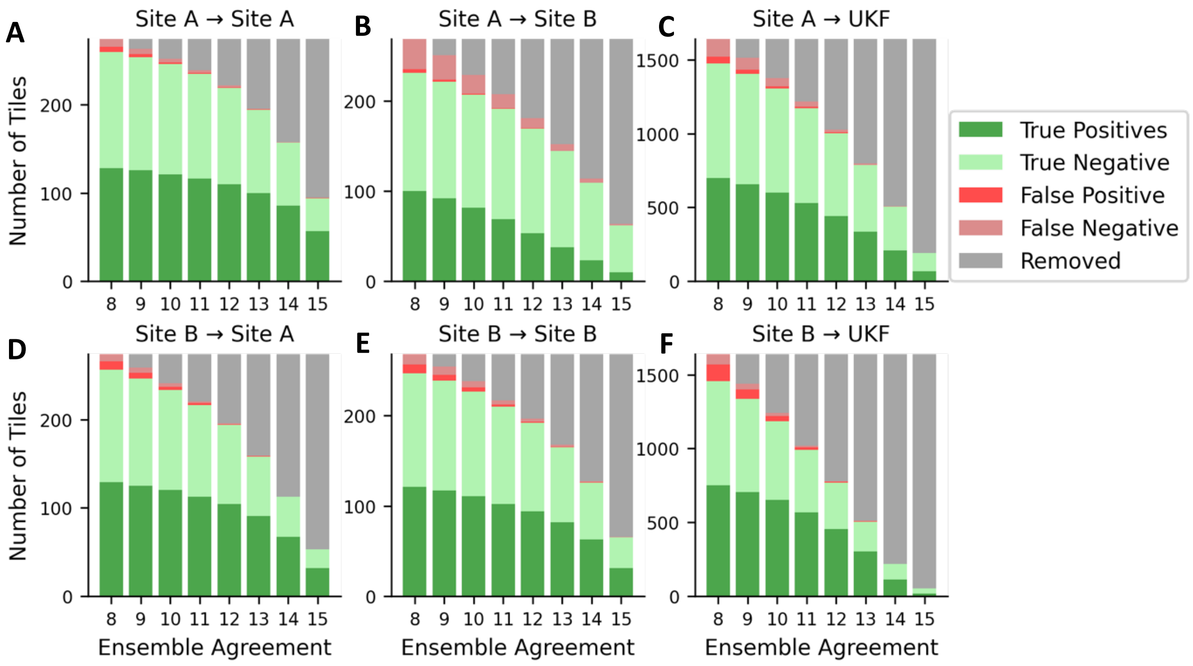

Supp Figure 15. Amount of TP, TN, FP and FN in tiles kept by the NoisyEnsemble. The NoisyEnsemble was trained with a noise level of 15% on **A-C.** data from site A or **D-F.** from site B. The bar charts depict the number of TP, TN, FP and FN predictions.

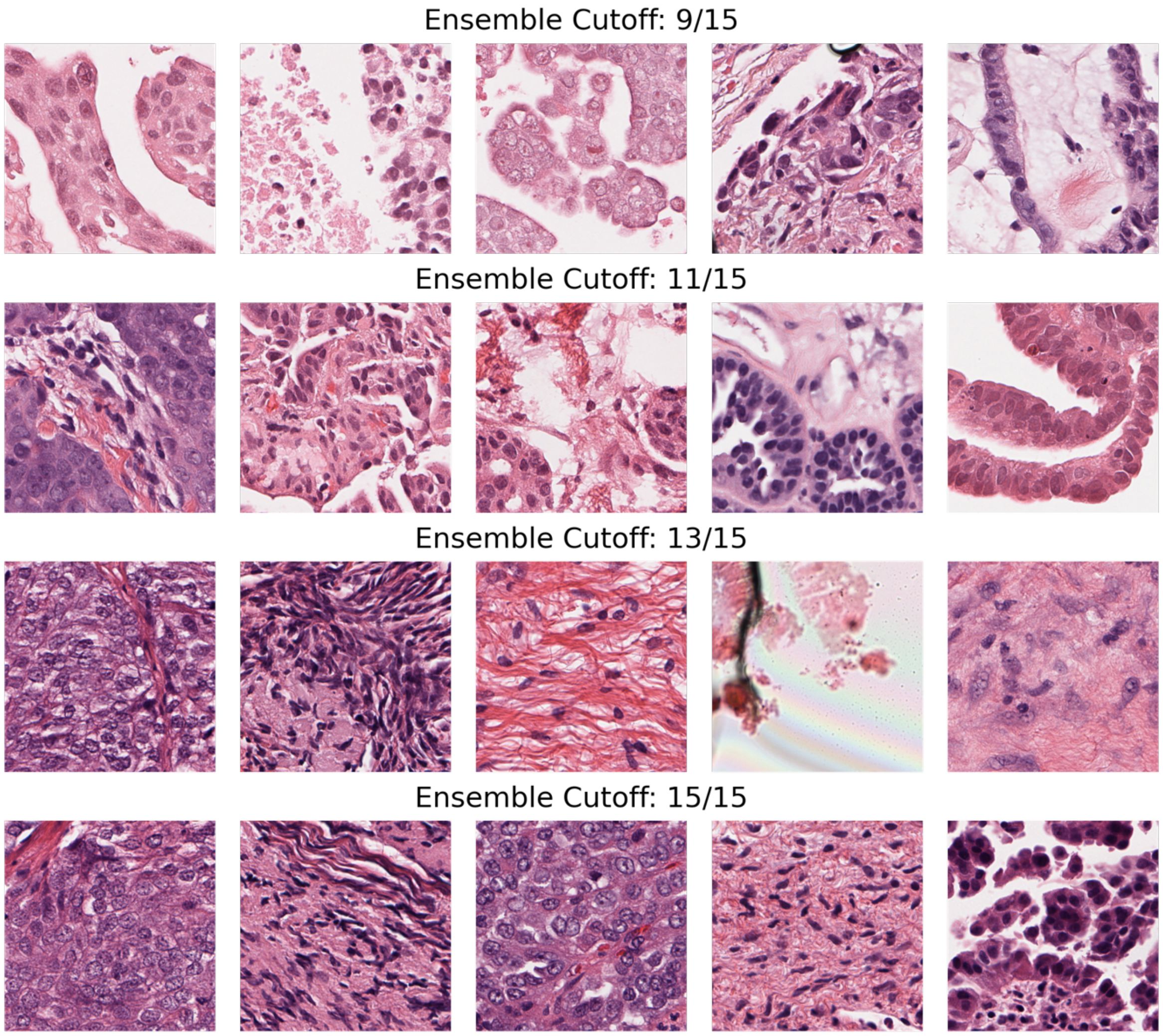

Supp Figure 16. Tiles excluded by thee NoisyEnsemble. The NoisyEnsemble (nolse level: 15%) was trained on data from dataset A and tested on data from dataset B. For the Ensemble agreement levels of 9/15, 11/15, 13/15 and 15/15 randomly 5 tiles are depicted which were removed by the ensemble at the respective agreement level.

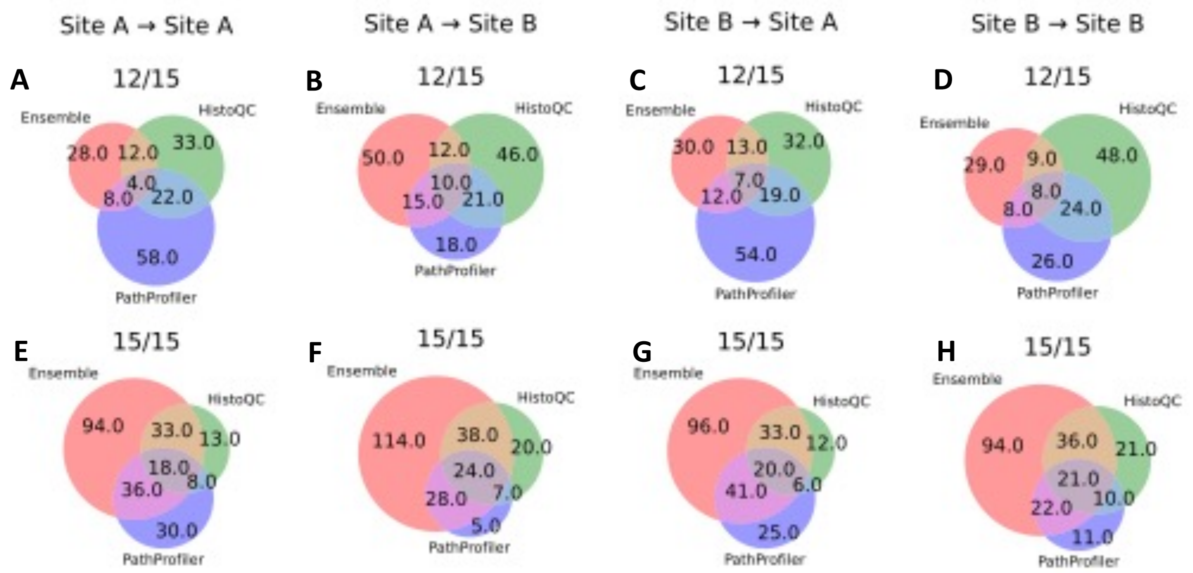

Supp Figure 17. Number of overlapping and uniquely removed tiles. Each circle of a Ven Diagrams contains the number of the tiles removed by the respective method (HistoQC, PathProfiler and NoisyEnsemble with noise level of 15% for an agreement level of **A-D.** 12/15 and **E-H.** 15/15), overlapping regions contain the number of images which were removed by several methods. Training and Testing dataset is indicated on the top of each column.

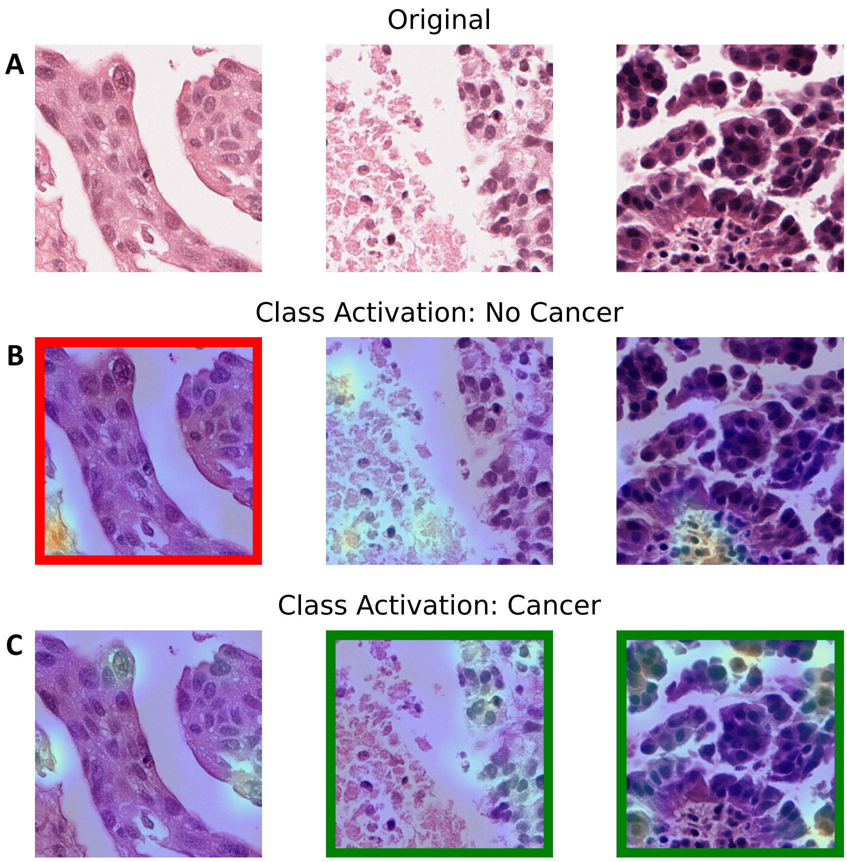

Supp Figure 18. Explainable AI for NoisyEnsembles. Grad-CAMs were calculated for every single cNN of the ensemble. Then the average over all individual Grad-CAMs was calculated to visualize the image regions which are important for the decision of the ensemble. **A.** three original image containing, all labeled as cancerous **B.** Grad-CAMs for the 'no cancer' prediction **C.** Grand-CAMs for the 'cancer' prediction. The actual prediction of our NoisyEnsemble is highlighted by the boxes (green… correct; red…wrong). One now sees that the network focuses on the correct parts of the image for its prediction. Especially in the first column, one can see that there is hardly any non cancerous tissue which is used this wrong prediction. The cancerous cells which are highlighted during the cancer prediction are in contrast really characteristic and pathologist could easily understand the basis for the decision.

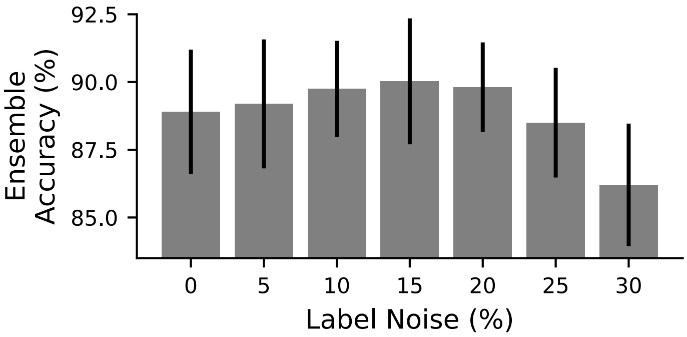

Supp Figure 19. Ensemble accuracies for combined test sets. In order to simulate the overall performance of the NoisyEnsemble trained with high quality data, we constructed one test dataset, containing 7 patients from site A, 7 patients from site B and 7 patients from the UKF.

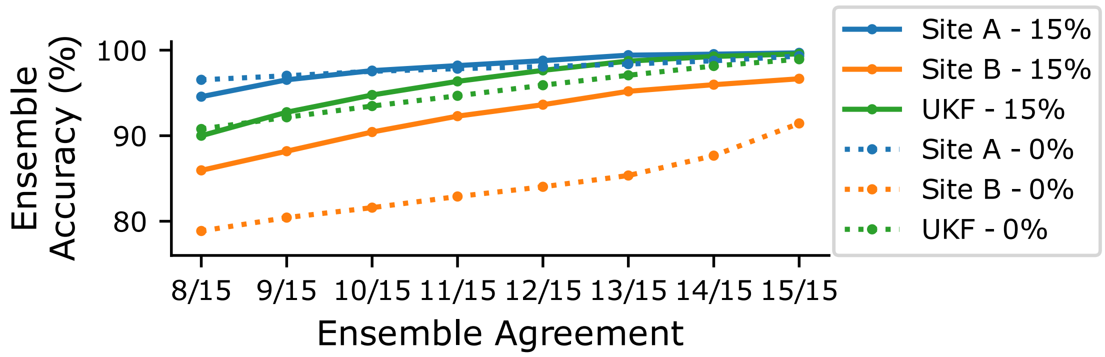

Supp Figure 20. Ensemble Accuracy for 0% and 15% noise. For high quality datasets ensembles trained without noise perform slightly better for the complete dataset (ensemble agreement 8/15), but this difference vanished when only secure predictions are taken into account. For low quality data (site B) the performance increase is clearly present for all levels of ensemble agreement.

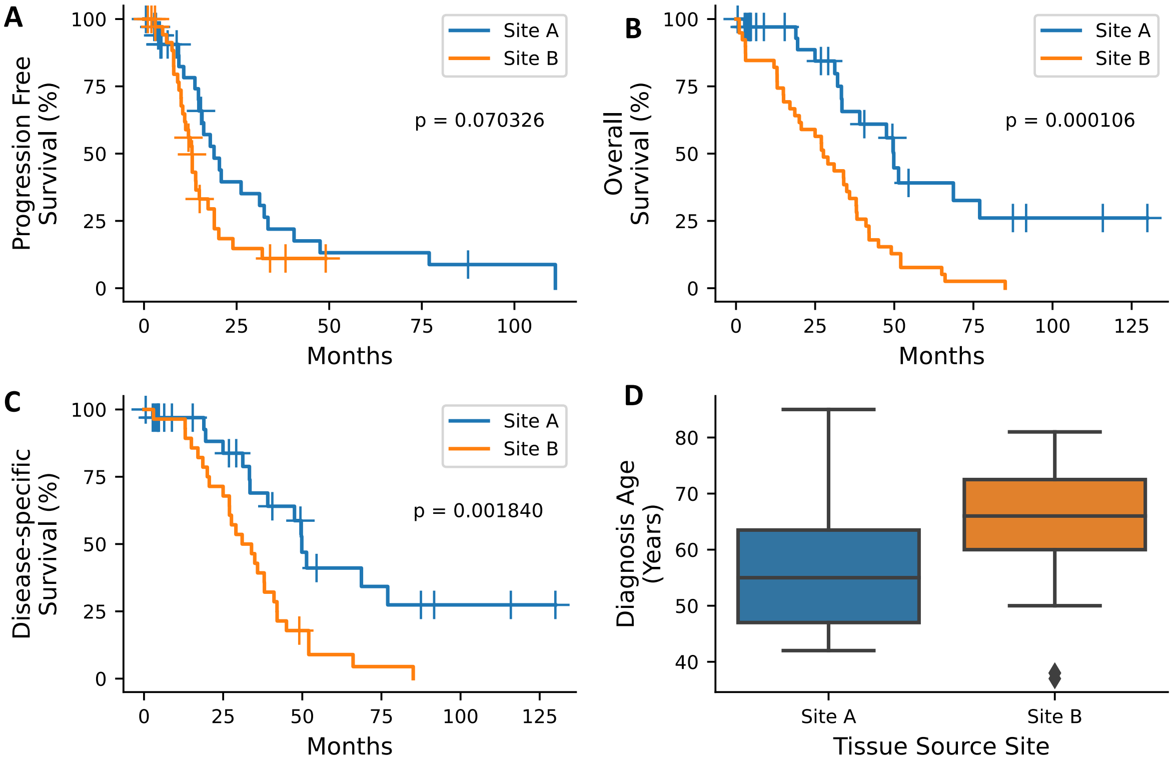

Supp Figure 21. Intrinsic differences between patients of source site A and B. Kaplan-Maier plots for **A.** Progression free survival, **B.** overall survival and **C.** disease specific survival. P-Values from the logrank test are indicated. **D.** Diagnosis age for the patients from source site A and B.

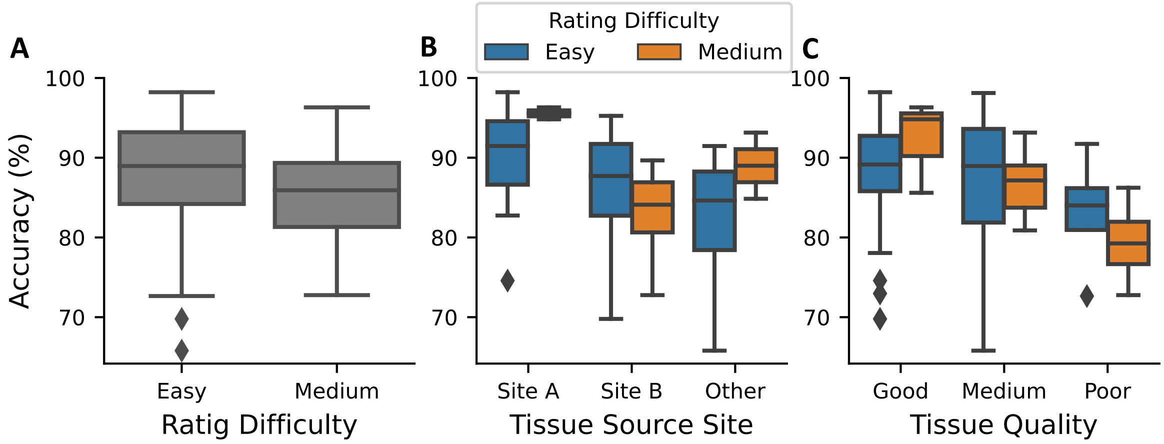

Supp Figure 22. Rating does not influence CNN performance. A pathologist scored all slides for their difficulty level in distinguishing cancer from non-cancerous areas, here called 'rating'. **A.** Accuracy for the different rating levels, no significant difference according to the t-test. **B.** Accuracy in dependance of tissue source site and rating. No significant differences were observed for the different rating levels within one source site. **C.** Accuracy in dependance of tissue quality (also assigned by a pathologist) and rating. No significant differences were observed for the different rating levels within one quality level.

Supp Table 1. Average performance for individual patients from the TCGA-OV dataset.

Patient ID	Tissue Source Site	Pathologist 1 Tissue Quality	Pathologist 2 Tissue Quality	Pathologist 1 Difficulty Rating	Accuracy	Precision	Recall	F1 Score
**TCGA-23-1111**	Site A	Good	Good	Easy	98.2	1.00	0.97	0.98
**TCGA-23-1123**	Site A	Medium	Medium	Easy	98.1	1.00	0.97	0.98
**TCGA-23-1109**	Site A	Medium	Good	Easy	98.1	0.99	0.97	0.98
**TCGA-23-1030**	Site A	Good	Good	Easy	97.6	0.97	0.99	0.98
**TCGA-23-1032**	Site A	Good	Good	Easy	97.1	0.95	1.00	0.97
**TCGA-23-1117**	Site A	Good	Medium	Easy	96.4	0.95	0.99	0.96
**TCGA-23-1022**	Site A	Good	Medium	Medium	96.3	0.98	0.95	0.96
**TCGA-25-1632**	Site B	Medium	Good	Medium	96.2	0.94	0.99	0.97
**TCGA-23-2077**	Site A	Medium	Good	Easy	96.1	0.99	0.93	0.96
**TCGA-23-1122**	Site A	Medium	Good	Easy	95.4	0.96	0.95	0.95
**TCGA-25-1329**	Site B	Good	Good	Easy	95.3	0.97	0.94	0.95
**TCGA-25-2396**	Site B	Good	Good	Easy	95.0	0.98	0.92	0.95
**TCGA-25-1870**	Site B	Medium	Good	Easy	94.9	0.97	0.94	0.95
**TCGA-23-2081**	Site A	Good	Medium	Medium	94.8	0.99	0.91	0.94
**TCGA-23-2079**	Site A	Good	Good	Easy	94.6	0.92	0.98	0.95
**TCGA-42-2589**	Other	Medium	Good	Medium	94.6	0.93	0.98	0.95
**TCGA-23-2649**	Site A	Good	Good	Easy	94.5	0.99	0.90	0.94
**TCGA-25-1635**	Site B	Medium	Medium	Easy	94.5	0.96	0.94	0.94
**TCGA-23-1120**	Site A	Good	Good	Easy	94.1	0.95	0.94	0.94
**TCGA-23-1114**	Site A	Medium	Medium	Easy	93.7	0.96	0.92	0.94
**TCGA-25-2401**	Site B	Medium	Medium	Easy	93.5	0.93	0.94	0.94
**TCGA-23-1113**	Site A	Good	Good	Easy	93.5	0.95	0.92	0.93

**TCGA-57-1994**	Other	Medium	Good	Medium	93.2	0.92	0.95	0.93
**TCGA-25-2398**	Site B	Medium	Good	Easy	93.0	0.97	0.89	0.92
**TCGA-23-1026**	Site A	Good	Medium	Easy	92.5	0.98	0.87	0.92
**TCGA-23-2084**	Site A	Good	Good	Easy	92.4	0.90	0.96	0.93
**TCGA-25-1322**	Site B	Poor	Medium	Easy	91.7	0.95	0.89	0.91
**TCGA-25-1315**	Site B	Medium	Good	Easy	91.7	0.94	0.90	0.91
**TCGA-23-2643**	Site A	Good	Good	Easy	91.7	1.00	0.84	0.91
**TCGA-3P-A9WA**	Other	Good	Good	Easy	91.5	0.90	0.95	0.92
**TCGA-23-1110**	Site A	Good	Good	Easy	91.5	0.90	0.94	0.92
**TCGA-25-1325**	Other	Medium	Good	Medium	91.2	0.99	0.84	0.90
**TCGA-25-1623**	Site B	Good	Good	Easy	91.0	0.97	0.85	0.90
**TCGA-WR-A838**	Other	Medium	Medium	Easy	90.9	0.87	0.98	0.92
**TCGA-23-2645**	Site A	Good	Good	Easy	90.8	0.99	0.82	0.90
**TCGA-25-2397**	Site B	Medium	Good	Easy	90.7	0.89	0.93	0.91
**TCGA-23-2078**	Site A	Good	Good	Easy	90.3	0.92	0.89	0.90
**TCGA-42-2582**	Other	Medium	Poor	Medium	90.2	0.92	0.90	0.90
**TCGA-23-2641**	Site A	Medium	Medium	Easy	89.8	0.90	0.90	0.89
**TCGA-23-1107**	Other	Good	Good	Medium	89.8	0.90	0.90	0.90
**TCGA-25-1324**	Site B	Medium	Good	Medium	89.7	0.88	0.93	0.90
**TCGA-23-2647**	Site A	Good	Good	Easy	89.3	0.91	0.89	0.89
**TCGA-23-1809**	Site A	Good	Good	Easy	89.3	0.92	0.87	0.89
**TCGA-59-A5PD**	Other	Medium	Good	Medium	89.3	0.90	0.91	0.89
**TCGA-23-1021**	Site A	Good	Good	Easy	89.0	0.84	0.97	0.90
**TCGA-57-1586**	Other	Medium	Good	Easy	89.0	0.95	0.83	0.88
**TCGA-23-1023**	Site A	Good	Good	Easy	88.4	0.90	0.87	0.88
**TCGA-25-1326**	Site B	Medium	Good	Medium	88.4	0.98	0.79	0.86
**TCGA-25-2409**	Site B	Good	Good	Easy	88.3	0.90	0.87	0.88
**TCGA-25-2400**	Site B	Good	Good	Easy	88.3	0.98	0.78	0.86
**TCGA-57-1992**	Other	Good	Medium	Easy	88.0	0.85	0.92	0.88
**TCGA-25-1878**	Site B	Medium	Good	Easy	87.8	0.89	0.88	0.87
**TCGA-25-2392**	Site B	Good	Medium	Easy	87.6	0.99	0.76	0.85
**TCGA-25-1321**	Site B	Medium	Poor	Medium	87.2	0.97	0.77	0.85
**TCGA-57-1993**	Other	Good	Good	Easy	86.7	0.90	0.84	0.86
**TCGA-23-1119**	Site A	Good	Medium	Easy	86.6	0.92	0.80	0.86
**TCGA-25-2404**	Site B	Medium	Medium	Easy	86.4	0.86	0.89	0.87
**TCGA-23-1027**	Site A	Good	Good	Easy	86.3	0.92	0.80	0.85
**TCGA-25-1323**	Site B	Poor	Medium	Medium	86.2	0.97	0.75	0.84
**TCGA-23-1121**	Site A	Good	Medium	Easy	85.8	0.94	0.77	0.83
**TCGA-23-2072**	Site A	Good	Good	Easy	85.7	0.90	0.81	0.84
**TCGA-25-1626**	Site B	Medium	Poor	Easy	85.6	0.89	0.82	0.85
**TCGA-25-2399**	Site B	Good	Good	Medium	85.6	0.88	0.83	0.85
**TCGA-57-1582**	Other	Good	Good	Easy	85.6	0.88	0.85	0.86
**TCGA-25-2390**	Other	Poor	Good	Medium	85.4	0.93	0.77	0.83
**TCGA-57-1585**	Other	Medium	Medium	Medium	84.9	0.86	0.85	0.85

**TCGA-42-2588**	Other	Medium	Good	Medium	84.4	0.87	0.82	0.83
**TCGA-23-1028**	Site A	Good	Good	Easy	84.4	0.89	0.80	0.83
**TCGA-25-1630**	Site B	Poor	Poor	Easy	84.3	0.93	0.75	0.82
**TCGA-25-2393**	Site B	Medium	Good	Easy	84.3	0.89	0.80	0.83
**TCGA-25-1625**	Site B	Medium	Good	Easy	84.2	0.88	0.81	0.84
**TCGA-42-2593**	Other	Poor	Medium	Easy	83.7	0.98	0.70	0.79
**TCGA-23-1031**	Site A	Good	Good	Easy	83.3	0.91	0.75	0.81
**TCGA-23-1116**	Site A	Good	Good	Easy	83.1	0.80	0.91	0.84
**TCGA-23-1029**	Site A	Good	Good	Easy	82.8	0.97	0.68	0.79
**TCGA-25-1634**	Site B	Medium	Medium	Medium	82.6	0.84	0.81	0.82
**TCGA-25-2391**	Site B	Medium	Good	Easy	82.3	0.90	0.74	0.80
**TCGA-13-A5FU**	Other	Medium	Poor	Easy	81.5	0.89	0.73	0.79
**TCGA-25-1871**	Site B	Medium	Good	Easy	81.5	0.85	0.79	0.80
**TCGA-25-2408**	Site B	Medium	Good	Easy	81.3	0.88	0.74	0.77
**TCGA-25-1328**	Site B	Medium	Good	Medium	80.9	0.89	0.71	0.78
**TCGA-25-1318**	Site B	Poor	Poor	Medium	80.6	0.91	0.70	0.77
**TCGA-25-2042**	Site B	Medium	Good	Easy	80.4	0.98	0.63	0.75
**TCGA-42-2590**	Other	Good	Good	Medium	78.9	0.75	0.91	0.81
**TCGA-57-1583**	Other	Medium	Medium	Easy	78.5	0.83	0.75	0.77
**TCGA-42-2591**	Other	Medium	Medium	Medium	78.1	0.83	0.73	0.76
**TCGA-5X-AA5U**	Other	Good	Good	Easy	78.1	0.96	0.57	0.69
**TCGA-25-1313**	Site B	Poor	Good	Medium	78.0	0.98	0.57	0.71
**TCGA-25-1631**	Site B	Medium	Good	Medium	76.3	0.90	0.60	0.71
**TCGA-25-1320**	Site B	Poor	Medium	Medium	75.8	0.73	0.85	0.78
**TCGA-42-2587**	Other	Medium	Good	Easy	75.0	0.71	0.94	0.80
**TCGA-23-1024**	Site A	Good	Good	Easy	74.6	0.81	0.65	0.72
**TCGA-25-1877**	Site B	Poor	Good	Medium	74.4	0.83	0.67	0.71
**TCGA-25-1627**	Site B	Good	Good	Easy	73.0	0.75	0.72	0.72
**TCGA-25-1628**	Site B	Poor	Good	Medium	72.8	0.73	0.76	0.73
**TCGA-25-1317**	Site B	Poor	Poor	Easy	72.7	0.91	0.51	0.65
**TCGA-25-1316**	Site B	Good	Good	Easy	69.8	0.71	0.74	0.70
**TCGA-13-A5FT**	Other	Medium	Good	Easy	65.8	0.93	0.35	0.47
**TCGA-FX-A3RE**	Other	Good	Good	Medium	62.2	0.61	0.78	0.67
**TCGA-VG-A8LO**	Other	Poor	Poor	Medium	59.2	0.59	0.71	0.63
**TCGA-25-1633**	Site B	Poor	Medium	Medium	56.9	0.72	0.26	0.34
